# Synthesis of Trimetallic Nanoparticle (NiCoPd)-Supported Carbon Nanofibers as a Catalyst for NaBH_4_ Hydrolysis

**DOI:** 10.3390/membranes13090783

**Published:** 2023-09-07

**Authors:** Ahmed Abutaleb, Ibrahim M. Maafa, Nasser Zouli, Ayman Yousef, M. M. El-Halwany

**Affiliations:** 1 Department of Chemical Engineering, Faculty of Engineering, Jazan University, Jazan 11451, Saudi Arabia; azabutaleb@jazanu.edu.sa (A.A.); imoaafa@jazanu.edu.sa (I.M.M.); nizouli@jazanu.edu.sa (N.Z.); 2 Department of Mathematics and Physics Engineering, Faculty of Engineering at Mataria, Helwan University, Cairo 11718, Egypt; 3 Department of Mathematics and Physics Engineering, Faculty of Engineering, Mansoura University, El-Mansoura 35516, Egypt

**Keywords:** chemical reaction engineering, sol-gel, electrospinning, catalytic nanofiber, trimetallic, hydrogen, sodium borohydride

## Abstract

The generation of H_2_ via the catalytic hydrolysis of sodium borohydride (SBH) has promise as a practical and secure approach to produce H_2_, a secure and environmentally friendly energy source for the foreseeable future. In this study, distinctive trimetallic NiCoPd nanoparticle-supported carbon nanofibers (NiCoPd tri-NPs@CNFs) is synthesized via sol-gel and electrospinning approaches. The fabricated trimetallic catalysts show an excellent catalytic performance for the generation of H_2_ from the hydrolysis of SBH. Standard physicochemical techniques were used to characterize the as-prepared NiCoPd tri-NPs@CNFs. The results show that NiCoPd tri-NPs@CNFs is formed, with an average particle size of about 21 nm. When compared to NiCo bimetallic NP @CNFS, all NiCoPd tri-NPs@CNFs formulations demonstrated greater catalytic activates for the hydrolysis of SBH. The improved catalytic activity may be due in the majority to the synergistic interaction between the three metals in the trimetallic architecture. Furthermore, the activation energy for the catalytic hydrolysis of SBH by the NiCoPd tri-NPs@CNFs was determined to be 16.30 kJ mol^−1^. The kinetics studies show that the reaction is of a first order with respect to the catalyst loading amount and a half order with respect to the SBH concentration [SBH].

## 1. Introduction

Hydrogen (H_2_) has garnered considerable attention as a potential viable fuel source for a variety of industrial operations in new energy vehicles [1,2,3]. Chemical H_2_ storage materials have received significant attention in recent years due to their potential to serve as an alternative source for producing H_2_ by releasing a substantial volume of hydrogen gas at room temperature [2,3,4]. Sodium borohydride (SBH, NaBH_4_) has a number of advantages over competing chemical H_2_ storage materials, such as ammonia borane and hydrazine hydrate, including the better regulation of the H_2_ generation rate (HGR) and purity, a safe manufacturing method, the ability to recycle the byproduct NaBO_2_ back into borohydride, and a lower temperature at which H_2_ may be liberated [2,5,6,7]. Moreover, it has superior physicochemical features, such as non-flammability, small molar mass (37.83 g mol^−1^), high H_2_ capacity (10.8 wt%), high solid-state stability at room temperature, and secure and reliable H_2_ production by hydrolysis. It is essential to use an efficient catalyst to generate a practical amount of H_2_ from SBH with an acceptable reaction rate. Catalysts for H_2_ production typically consist of noble metal nanoparticles (NPs) [8,9,10,11,12]. However, the high cost of their production and restricted availability hamper their eventual industrial uses. As an alternative, inexpensive transition-metal NPs, including iron, cobalt, and nickel, have been used in recent years for H_2_ production from SBH [13,14,15,16]. It has been suggested that bi- and trimetallic NPs possess higher catalytic activities in various chemical processes than their counterparts due to such lattice geometric, strain, and electronic charge transfer influences [2]. In addition, it is also believed that the alloying of noble metals with other non-noble transition metals results in increasing the catalytic performance and reducing the overall cost of the process [16]. For instance, Jiang et al. [17] found that bimetallic AgCo alloy catalysts displayed a five-times-better catalytic performance than Co catalysts in the hydrolysis of SBH. Bimetallic synthesis and catalytic activity have received a lot of attention compared to trimetallic ones during H_2_ production from SBH. It is noteworthy to highlight that the amount of research on the latter has been steadily increasing in the recent years. In light of the fact that the synergistic interaction of multiple components has an impact on the catalytic performance, attempts have been conducted to synthesize trimetallic catalysts in the hopes of improving the catalytic activities of the hydrolysis of SBH [18]. For instance, Wang et al. [18] synthesized 2D CuCoNi nanosheets through an in situ reduction using SBH. The prepared catalyst exhibited a superior catalytic performance and great stability to SBH hydrolysis compared to bimetallic NPs. It demonstrated a 1.3-fold higher catalytic activity towards H_2_ release from SBH than the bimetallic CuCo alloy. Moreover, the separation process of the solid catalyst from the liquid reaction solution was easy. In fact, having two magnetic elements, Co and Ni, makes the catalyst practical recycling process more suitable. Patil et al. [19] fabricated an iron–cobalt–copper trimetallic oxide catalyst via the combustion synthesis process. The synthesized catalyst demonstrated effective catalytic activity for H_2_ release from SBH. The maximal rate of H_2_ generation was 1380 mL min^−1^ g^−1^, while the rates for Fe, Co, and Cu oxides were 965, 226, and 126 mL min^−1^ g^−1^, respectively, whereas bimetallic FeCu, CuCo, and FeCo oxides had values of 861, 784, and 756.3 mL min^−1^ g^−1^, respectively. In this way, the high HGR was achieved by the synergistic action of the three metals in the FeCuCo trimetallic oxide. The catalytic performance of the catalyst was demonstrated to be superior for eight cycles. Jiao et al. [2] prepared two different compositions of colloidal Ni/Au/Co trimetallic NPs shielded by PVP used in the in situ co-reduction of metal ions via SBH. The catalytic performance of prepared trimetallic formulations were compared to that of their previously reported bimetallic NiAu [20] in the H_2_ production via the hydrolysis of SBH. The prepared trimetallic NPs (Ni_50_Au_10_Co_40_) demonstrated the HGR was 790 mol-H_2_ per h per mol-M, while Ni_50_Au_50_ produced 800 mol-H_2_ per h per mol-M. The activity of the trimetallic compound was lower than that of the bimetallic alloy. It should be noted, however, that trimetallic NPs (Ni_50_Au_10_Co_40_) are much more cost-effective catalysts for hydrogen generation from SBH than bimetallic NPs (Ni_50_Au_50_) when accounting for the Au%. Khan et al.’s [21] stepwise metal-displacement plating technique was applied to produce Cu^0^-based NPs, Cu-Ag-Ir, Cu-Pd-Ir, and Cu-Ag-Pd. The fabricated catalysts were used to produce H_2_ from hydrazine hydrogen storage material. The trimetallic (Cu-Pd-Ir, Cu-Ag-Ir, and Cu-Ag-Pd)) catalysts showed better catalytic activates than the bimetallic (Cu-Ag, Cu-Ir, and Cu-Pd) catalysts because of the three metals’ synergistic effects and electron interactions. The results prove that the existence of an appropriate third noble metal may result in an increase in the catalytic activity of the Cu-based bimetallic alloys. Since metal NPs have a high surface energy and a magnetic attraction to each other, they tend to aggregate, which can decrease their catalytic activity and shorten the lifetime of catalysts. In this context, the depositing of metal NPs on the matrix of supporting materials with higher specific surface areas (e.g., graphene, carbon-based materials, metal oxides, metal–organic framework, polymer, etc.) can maximize the dispersion of metal NPs without aggregation and may be an appropriate strategy to enhance the characteristics of metal NP catalysts [22,23,24,25,26,27,28,29,30,31,32,33]. Our group reports the effect of using carbon nanofibers (CNFs) as an efficient catalytic support matrix for mono- and bimetallic NPs in the H_2_ production from SBH and ammonia borane [7,34,35]. Here, the electrospinning technique is applied to decorate CNFs with trimetallic Ni-Co-Pd NPs for H_2_ production from SBH hydrolysis. The NiCoPd trimetallic NPs@CNFs are prepared using sol-gel and electrospinning approaches. To evaluate the catalytic activity of the synthesized NiCoPd trimetallic NPs@CNFs, it is compared with NiCo bimetallic NPs@CNFs under the same reaction conditions. This study shows that as-prepared trimetallic NPs possess a significantly higher catalytic activity for SBH hydrolysis than the comparable bimetallic NPs. The constructed catalytic trimetallic NFs are extremely stable toward H_2_ production from SBH for 10 cycles.

## 2. Experimental

### 2.1. Materials

Sodium borohydride (NaBH_4_, SBH), cobalt (II) acetate tetrahydrate (CoAc), nickel (II) acetate tetrahydrate (NiAc), palladium (II) acetate (PdAc, 99.9%), and polyvinylpyrrolidone (PVP, average Mw~1,300,000) were used. N, N, Dimethylformamide (DMF) and acetone were used as solvents. All chemicals were purchased from Aldrich Co., St. Louis, MO, USA.

### 2.2. Preparation of the NiCoPd tri-NPs@CNFs Catalyst

A total of 1.5 g of PVP was stirred into a solution of 10 mL of ethanol until it was completely dissolved. PVP was added to the solvent very slowly. Thereafter, trimetallic precursors were added to the polymer solution. PVP solutions containing trimetallic acetates of four distinct compositions were produced. After including trimetallic acetates, the sol-gel underwent a noticeable color shift. At a temperature of 50 °C, the produced sol-gels were mixed for three hours. The subsequent step was electrospinning with a lab-sized spinner and a high voltage (18 KV). The homemade glass enclosure protected the spinning system from its surroundings. The gap between the negative and positive electrodes was 18 cm. The flow rate of the sol-gels was 0.9 mL/h. This spinning operation of sol-gel was completed at an ambient temperature. Electrospun NF mats were formed and then peeled off from a plastic substrate. They were subsequently vacuum dried at 60 °C for 12 h. Lastly, they were carbonized at 850 °C for 3 h under vacuum with a constant flow of Ar gas. The heating rate was 3 °C min^−1^. The bimetallic product was also produced using the same procedures.

### 2.3. Characterization

The characterizations of the as-prepared NiCoPd tri-NPs@CNFs catalyst were performed using the identical standard technique presented in our recent publications [7,28,36,37].

### 2.4. Hydrolysis of SBH Using the NiCoPd tri-NPs@CNFs Catalyst

**Scheme 1:**
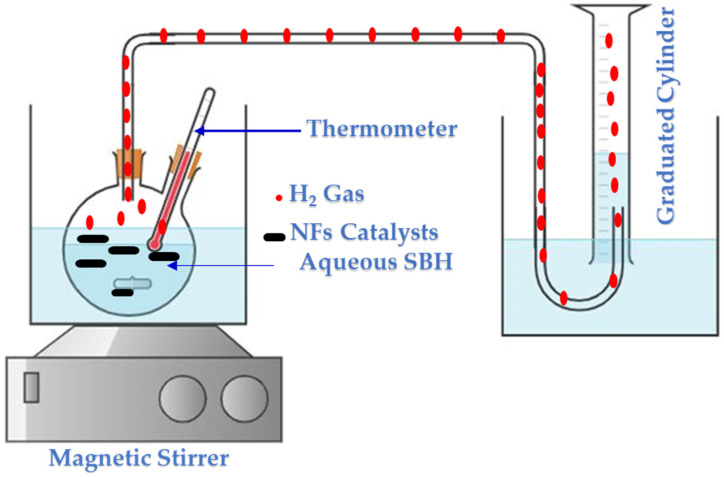
Experimental set-up for H_2_ generation.

A round-bottomed flask with two necks, one of which was sealed with a stopper and the other of which was attached to a gas burette, contained the SBH solution and the catalyst (Scheme 1). To regulate the reaction temperature, this device was immersed in a water bath. In order to manage the temperature of the reaction, a thermocouple was used. The reactions were initiated by adding 1 mmol of alkaline SBH and 0.05 g mg of catalyst to a flask, followed by magnetic stirring at 1000 rpm at 25 °C. The volume of gas produced was measured with a gas burette using the water displacement technique. The hydrogen that was evolved was plotted against the duration of time that passed. When no hydrogen gas was being produced, the process was stopped. The identical method was conducted without the addition of any catalytic material to serve as a control experiment. All of the synthesized catalysts were put through the same rigorous testing procedures. To further examine the kinetics of SBH hydrolysis, the reaction was run at several doses of catalyst, SBH, and temperatures (from 298 to 313 K). The effectiveness of recycling the proposed NFs was also evaluated. In order to assess the catalyst’s durability, this process was repeated multiple times using the same set of catalytic NFs. For each cycle, we used 1 mmol of SBH, 50 mg of catalyst, 25 °C, and 1000 rpm.

## 3. Results and Discussion 

### 3.1. Characterization of Pd-NiCo@CNFs

Electrospinning has been widely acknowledged as a straightforward and productive method for producing nanofibers (NFs) from a wide range of polymers. A sol-gel can be created by mixing the polymer solution with dissolved metal salt, which can be used to produce functional metals. Due to their high polycondensation propensity [29], acetates have found widespread use as metal precursors. For the formation of the sol-gel network, it is imperative that the solution components exhibit complete miscibility and endure polycondensation, as demonstrated in Equation (1):

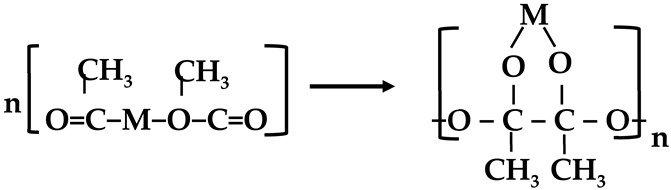
(1)
where M is the metals [38].

**Figure 1:**
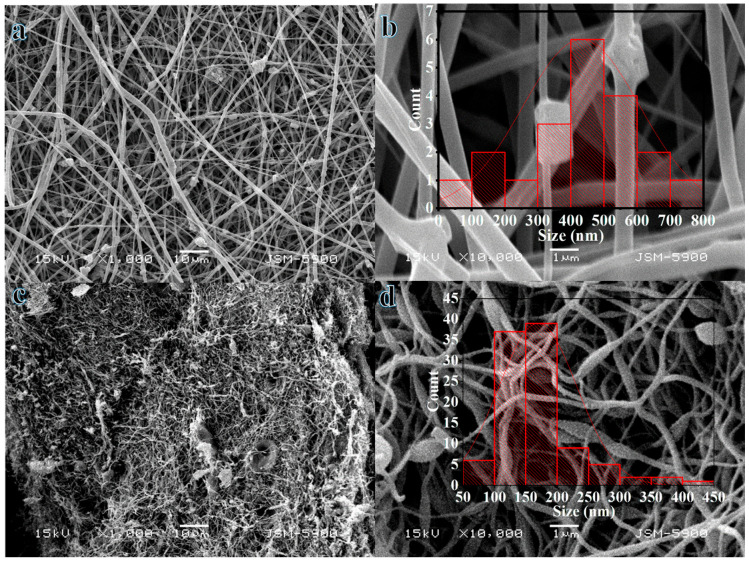
Low- (**a**) and high (**b**)-magnification SEM images of the electrospun NF materials consisting of CoAC, NiAc, and PdAc/PVP sol-gels after vacuum drying (**a**,**b**) and their obtained powder after calcination under vacuum in Ar atmosphere at 800 °C (**c**,**d**).

**Figure 2:**
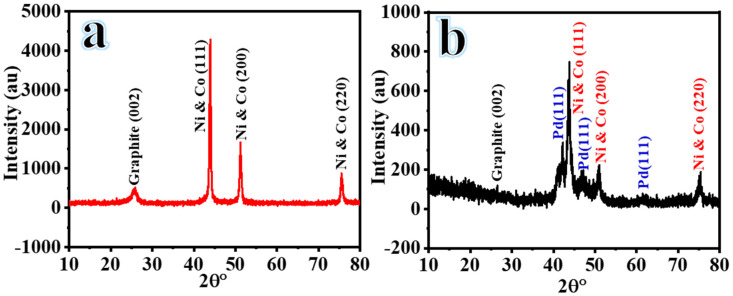
XRD analysis of the obtained powder after calcined electrospun NF mats composed of PVP, CoAC, and NiAc (**a**), and electrospun NF mats composed of PVP, CoAC, NiAc, and PdAc at 800 °C (**b**).

**Figure 3:**
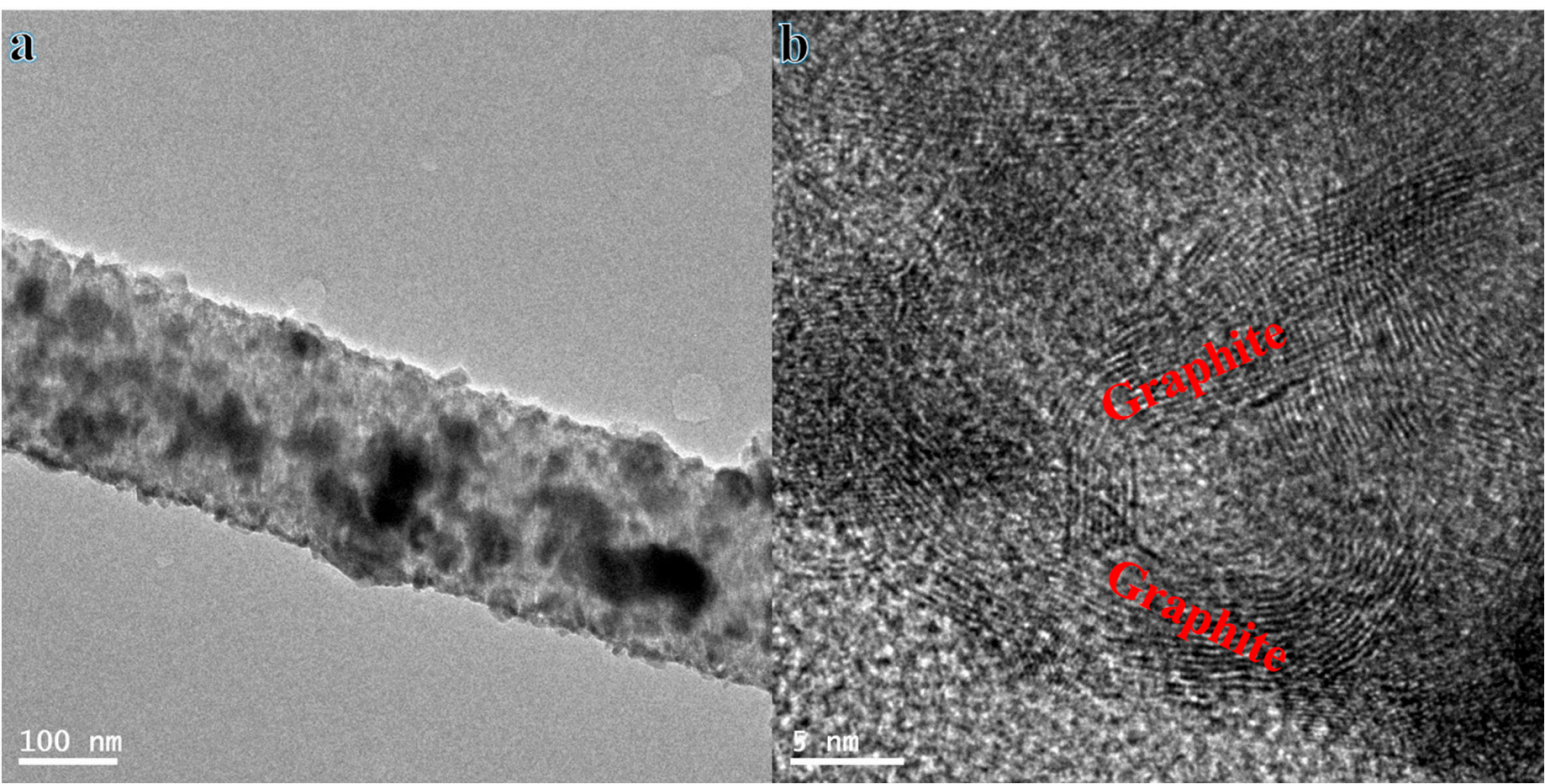
TEM (**a**) and HR TEM (**b**) images for the obtained Pd-decorated NiCo@ CNFs.

**Figure 4:**
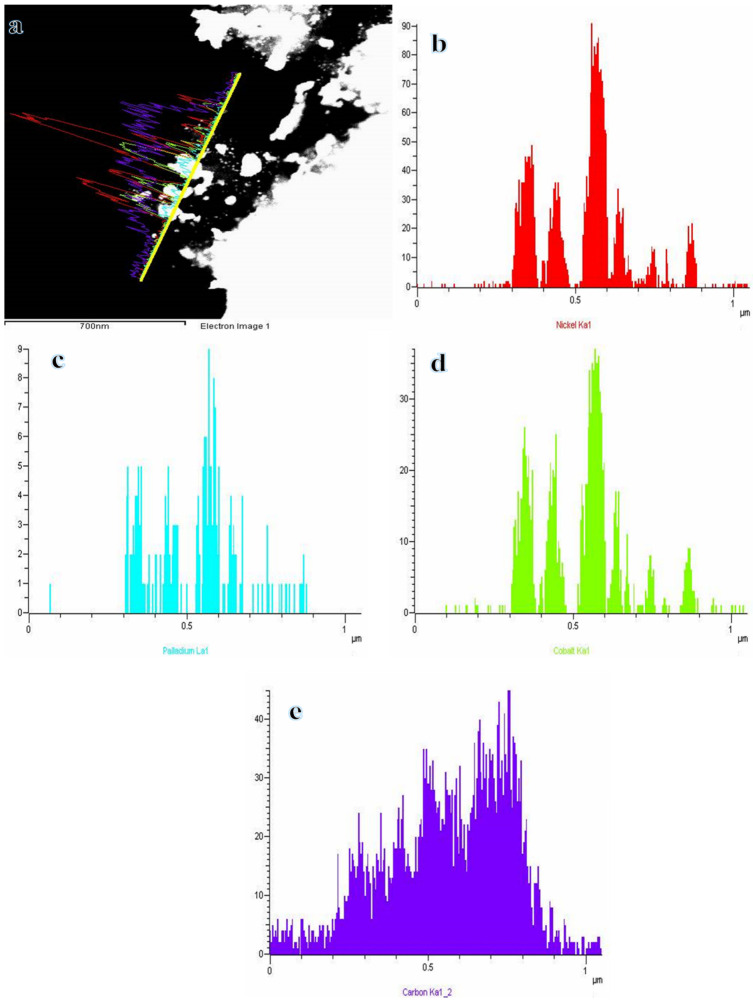
STEM image (**a**) for a single calcined nanofiber along with the line TEM EDX analysis for Ni (**b**), Pd (**c**), Co (**d**), and C (**e**) for the obtained Pd-decorated NiCo@ CNFs.

The literature suggests that PVP/metal acetate combinations exhibit an excellent nanofibrous structure. According to the literature, electrospinning is the most common method used for producing functional inorganic NFs. The Ni_0_._2_Co_0_._5_Pd_0_._2_ tri-NPs@CNFs catalyst with the best catalytic performance was completely characterized. Both low- and high-magnification SEM images of the resultant NF mats after electrospinning a polycondensate sol-gel consisting of CoAc/NiAc/PdAc/PVP are shown in Figure 1a,b, respectively. The conclusion that can be drawn from the figure is that the combination of the acetates does not have an effect on the structure as the NFs with a smooth and excellent architecture are produced without any beads being noticed. The average size of the produced NFs is ~431.7 nm (inset: Figure 1b). The SEM images of carbonized electrospun NF mats at 850 °C in an Ar atmosphere is shown in Figure 1c,d. As shown in Figure, a good nanofibrous morphology and continuous NFs are preserved after calcination. The proposed calcination strategy did not have an effect on the nanofibrous morphology. The mean size reduced to an extremely small 169.4 nm (inset: Figure 2b). The diameters reduced because the polymer and acetates used to produce them was decomposed and eliminated. XRD is a reliable method for examining inorganic substances. The XRD analysis pattern of the sintered PVP, CoAc, and NiAc electrospun NF mats is shown in Figure 2a. Diffraction peaks at 2Ɵ of 43.78°, 51.17°, and 75.19° were observed; these angles are typical of (111), (200), (110), and (021) crystal orientations, respectively. These planes are consistent with the synthesis of those of the cubic crystalline Co (JCDPS card number 15-0806) or crystalline Ni (JCPDS card number 04-085) or both [39]. The following are some possible explanations for this: in the periodic table, nickel and cobalt are located close to one another; (ii) their atomic weights are quite close to one another (Ni: 58.7 and Co: 58.9), making their atoms very similar in size; (iii) the findings of the XRD experiment confirm that both metals have FCC crystal structures with about the same cell parameters, (Co) 3.544 and (Ni) 3.523); and (iv) the two metals have the same valence. As a result, these two metals can combine to produce a substitutional alloy. That is to say, atoms of nickel can be substituted for atoms of cobalt in an FCC cobalt crystal, and vice versa. Due to the high melting points of Co (1495 °C) and Ni (1453 °C), no metal vaporizes during the carbonization process, despite the fact that a significant quantity of both precursors were used in the initial electrospun solution. This means that the resulting NFs are composed of pure CoNi metals with an FCC crystal structure. The Scherrer’s equation was used to determine the average grain size of Ni, Co, and Pd, which was found to be about 27 nm. If there was no catalyst present to catalyze the graphitization reaction, calcination in an argon environment caused practically all of the PVP to completely vanish. The existence of a single peak at 26.5° coincided with an observed d spacing of 3.37 A, resulting in the formation of graphite-like carbon ((002), JCPDS: 41-1487), confirming the graphitization of PVP in the presence of the trimetallic alloy [40]. The results from the XRD analysis of calcined PVP, CoAc, NiAc, and PdAc electrospun NF mats are shown in Figure 2b. The spectra support the development of cubic palladium (Sp.gr Fm3m (225)) at 2Ɵ of 42.19°, 46.85°, and 64.82°, which agree with the (111), (200), and (220) crystal planes, respectively, and are consistent with the initial composition of the electrospun mats [41]. The main grain size was estimated to be 21 nm using Scherrer’s equation. The existence of the weak intensity peak of graphite indicated the very slight presence of carbon. Barakat et al. [41] revealed that the good graphitization of PVA could be achieved during the calcination of electrospun CoAc/PVA. However, because of the presence of Pd NPs, the graphitization of PVA could be eliminated using electrospun Pd NPs-CoAc/PVA. In addition to this, it appeared that the process of graphitization was not the one that was catalyzed by the Pd, but rather the disintegration of PVA into compounds with a low molecular weight. The TEM picture of the obtained nanofibers is shown in Figure 3. Normal TEM images (Figure 3a) show that metallic NPs are randomly dispersed around NFs, suggesting that the resulting product is Ni-Pd-Co-@CNFs. The HR TEM picture in Figure 3b shows the thin layer of carbon with good crystallinity that is produced, which may boost adsorption and electric conductivity; in other words, it provides very good electron transportation. As can be seen in Figure 3a, the associated metallic NPs are likewise dispersed throughout the thin layer of CNFs. Linear analysis TEM EDX was used to investigate the Ni, Co, and Pd distributions throughout the length of the formed NFs (Figure 4). Figure 4a shows that metallic NPs are distributed along the chosen line. Remarkably, the distribution curves for both Ni (Figure 4b) and Co (Figure 4d) metals are identical, indicating that they are intermixed at the crystalline level. On the contrary, the spectral evidence points to Pd (Figure 4c) as the doping element, which indicates the formation of Pd-NiCo. Carbon is the outermost component of the prepared NFs (Figure 4e). This suggests that the CNFs formed a protective layer around the metal NPs. It may be easy for CNFs to adsorb SBH and transfer electrons, both of which contribute to it being easy to separate H atoms.

### 3.2. Catalytic Hydrolysis of SBH

**Figure 5:**
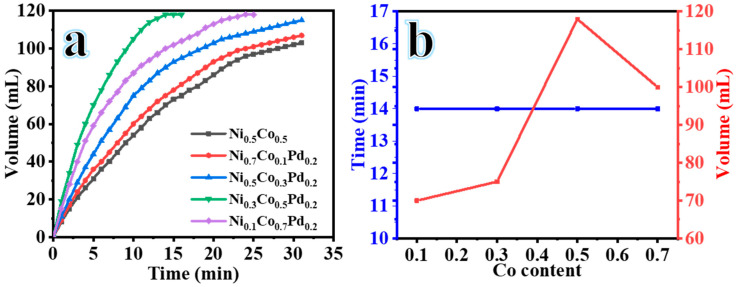
H_2_ production volume in terms of time by hydrolysis of SBH using different compositions of NiCoPd@CNFs (**a**) and the effect of the increase in Co content in NiCoPd@CNFs on the hydrolysis of SBH (**b**) at 25 °C [SBH] = 1 mmol and 1000 rpm.

The hydrolysis of SBH was accomplished using the as-prepared NiCoPd tri-NPs@CNFs catalyst. Different catalytic activities were shown when the catalyst’s composition was altered. All Ni/Co/Pd@CNFs demonstrated good catalytic performances towards H_2_ generation from SBH, as shown in Figure 5a, albeit having somewhat varying generation rates. When compared to other formulations, the Ni_0.3_Co_0.5_Pd_0.2_ tri-NPs@CNFs catalyst demonstrated the highest catalytic activity. The highest generated hydrogen yields and HGR were calculated from the hydrolysis of 1 mmol SBH at 25 °C using 0.05 g catalysts with different Ni/Co/Pd ratios, and the results are displayed in Table 1. In addition, the produced NiCoPd tri-NPs@CNFs showed enhanced H_2_ production activity compared to the bimetallic NiCo@CNFs (Table 1). The interpretation points to the synergistic impact between the three metals in NiCoPd tri-NPs@CNFs as the likely cause of the high HGR. Increases in H_2_ production were also seen when the ratio of Co to Ni in the catalyst was increased (Figure 5b), which could be attributed to the superior activity of Co NPs compared to Ni NPs in H_2_ production from SBH.

**Scheme 2:**
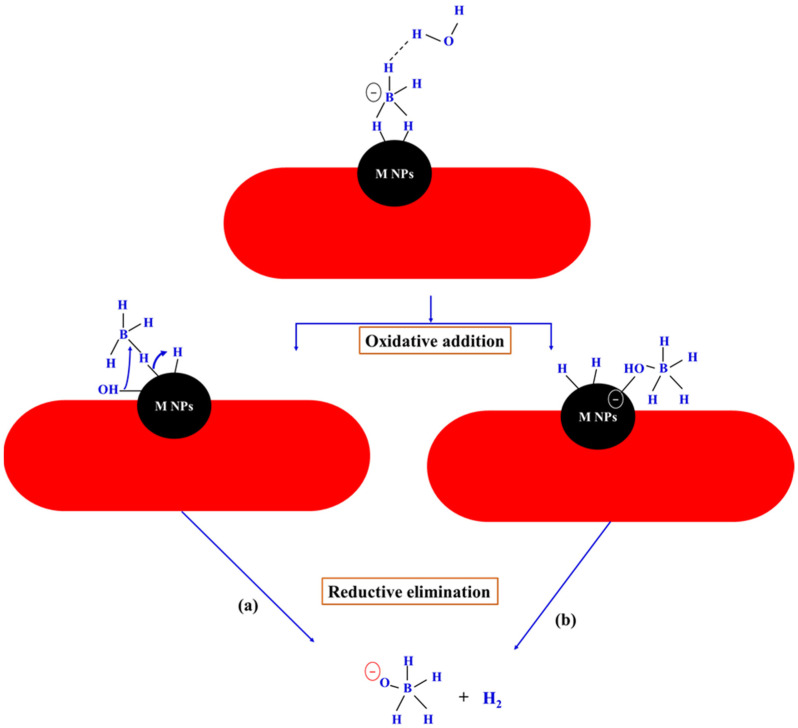
Suggested mechanism of H_2_ production using NiCoPd tri-NPs@CNFs.

The compound SBH functioned as a hydrogen donor, providing one of the two hydrogen atoms in the resulting H_2_ molecule. The second hydrogen atom was obtained from a proton originating from H_2_O [42,43]. The rate-determining step was the activation of one O–H bond in the adsorbed water. Oxidative addition can potentially occur through the facilitation of a hydrogen-bonding interaction between a proton from H_2_O and a surface-coordinated BH4^−^ in [BH3–H–H–OH]^−^. This interaction aided the oxidative addition process by decreasing the electron density of the O–H bond. Moreover, the transfer of the negative charge of BH3^−^ to H_2_ was facilitated by the excellent conductivity of the CNF substrate [44]. Ultimately, H_2_ was liberated from the surface of the catalyst through either reductive elimination (Scheme 2a) or a concerted σ-bond metathesis-like process involving a surface-coordinated BH4^−^ and a hydrogen atom derived from H_2_O, likely facilitated by a surface hydroxide ion (OH^−^) (Scheme 2b) [45].

## 4. Influences of Catalyst Dose

**Figure 6:**
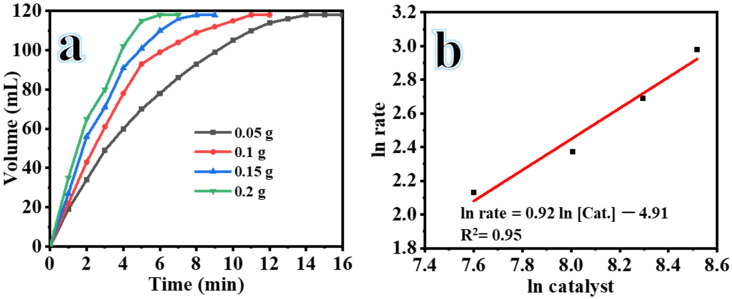
H_2_ production volume versus time from hydrolysis of SBH using different catalyst loads of Ni_0.3_Co_0.5_Pd_0.2_@CNFs at 25 °C, [SBH] = 1 mmol, and 1000 rpm (**a**), and logarithmic plot of the H_2_ production rate versus catalyst loading amount (**b**).

HGR from the hydrolysis of SBH as a function of catalyst concentration is shown in Figure 5. All other conditions (i.e., SBH = 1 mmol at 25 °C and a stirring speed of 1000 rpm) were held constant, and Ni_0.3_Co_0.5_Pd_0.2_ tri-NPs@CNFs were utilized at quantities of 0.05, 0.1, 0.15, and 0.2 g. The effect of various amounts of Ni_0.3_Co_0.5_Pd_0.2_ tri-NPs@CNFs on the developed H_2_ is depicted in Figure 6a. The rate at which H_2_ was produced increased gradually as the catalyst loading was increased (Table 2). This may have occurred because there were more surface active sites available, allowing SBH hydrolysis to proceed more rapidly [46,47,48]. Overall, the results show that the rate at which hydrogen is produced from SBH hydrolysis is proportional to the loading of the Ni_0.3_Co_0.5_Pd_0.2_ tri-NPs@CNFs catalyst. However, it is well known that, for any given catalytic reaction, an efficient operation at a low catalyst loading level is always the best. The logarithmic plot of HGR versus the sloped line depicting the catalyst amount is 0.92 in Figure 5b. The hydrolysis of SBH proceeds according to 1st-order kinetics with regard to Ni_0.3_Co_0.5_Pd_0.2_ tri-NPs@CNFs dosage. It has been reported elsewhere [49] that more catalysts result in more H_2_ being produced because they enhance the competition for the same quantity of SBH.

## 5. Influences of SBH Concentration 

**Figure 7:**
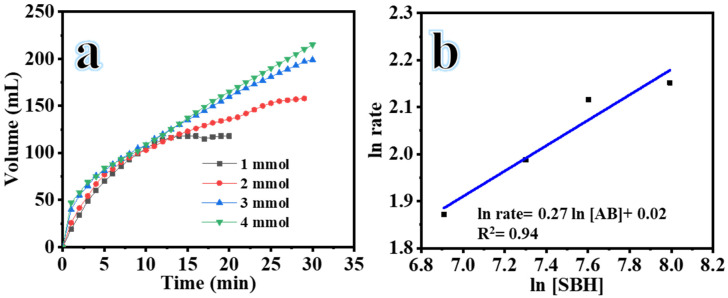
H_2_ production volume versus time from hydrolysis of SBH using different [SBH] levels at 25 °C, Ni_0.3_Co_0.5_Pd_0.2_@CNFs amount = 0.05 g, and 1000 rpm (**a**), and logarithmic plot of H_2_ production rate versus [SBH] (**b**).

The HGR relies considerably on the SBH. The effects of 1–4 mmol SBH were examined while keeping all other variables constant (0.05 g of Ni_0.3_Co_0.5_Pd_0.2_ tri-NPs@CNFs, 25 °C, and 1000 rpm stirring rate). The volume of H_2_ produced was found to gradually increase as the SBH concentration increase (Figure 7a). The rate at which H_2_ was produced, however, remained practically unchanged, regardless of the concentration of SBH present. The logarithmic plot of HGR versus catalyst concentration is plotted in Figure 7b, and the line slope is 0.27. These results show that the Ni_0.3_Co_0.5_Pd_0.2_ tri-NPs@CNFs catalyzed hydrolysis of SBH follows a 0.27-order kinetics with respect to the change in SBH concentration. This was due to the use of a low SBH concentration, as higher concentrations resulted in the formation of sodium metaborate that both increased the viscosity and slowed down the rate of the reaction [50,51,52]. A zero-order reaction was observed at higher concentrations than those explored in our investigation.

## 6. Influences of Reaction Temperature

**Figure 8:**
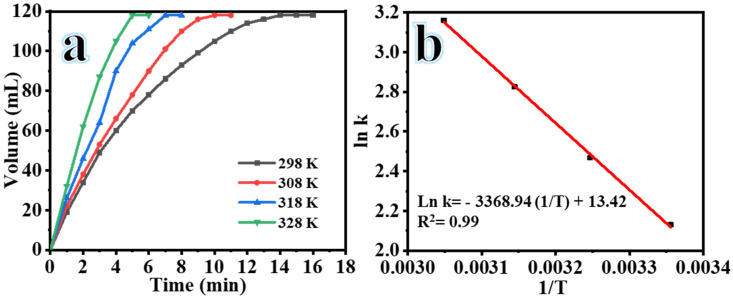
Influence of reaction temperature on the hydrolysis of SBH catalyzed by Ni_0.3_Co_0.5_Pd_0.2_@CNFs (**a**) and the Arrhenius plot of ln rate versus 1/T (**b**) used ([SBH] = 1 mmol, Ni_0.3_Co_0.5_Pd_0.2_@CNFs = 0.05 g, and 1000 rpm).

The rate at which SBH is catalytically hydrolyzed to generate H_2_ is highly influenced by the reaction temperature (T) [46,47,48]. By changing the (T) from 25 to 55 °C, the effect of (T) on Ni_0.3_Co_0.5_Pd_0.2_ tri-NPs@CNFs stimulated SBH hydrolysis was studied, while all other parameters were held constant (0.05 g of Ni_0.3_Co_0.5_Pd_0.2_ tri-NPs@CNFs, 1 mmol of SBH, and a stirring speed of 1000 rpm). Figure 8a displays the dramatic acceleration of hydrogen production with increasing (T). The Ni_0.3_Co_0.5_Pd_0.2_ tri-NPs@CNFs catalyst showed initial activity at 25 °C, displaying an HGR of 8.43 mL min^−1^ (118 mL in 16 min). H_2_ could be produced at a higher rate thanks to the high number of active sites in the catalyst, which could effectively activate the SBH reactant and the water molecule [19]. The HGR increased sharply to 11.80 mL min^−1^ upon increasing the (T) to 35 °C, and the process reached equilibrium in just 11 min. By applying heat, the efficient interaction between the SBH reactant, water, and Ni_0.3_Co_0.5_Pd_0.2_ tri-NPs@CNFs catalyst was sped up, which increased the reaction rate [19]. The HGR increased from 16.9 mL min^−1^ (118 mL in 7 min) to 23.6 mL min^−1^ (118 mL in 5 min) when the (T) was increased from 45 to 55 °C, respectively. According to the results, the HGR increases proportionally with increases in the (T), indicating that the process follows first-order kinetics. In Figure 5b, the Arrhenius equation is used to derive the activation energy (Ea) from the initial rate of produced H_2_. HGR is related to (T) and (Ea) according to the Arrhenius equation [46,47,48]. The reaction appears to follow 1st-order kinetics with regard to the reaction temperature, as indicated by the straight line (Figure 8b). Ni_0.3_Co_0.5_Pd_0.2_ tri-NPs@CNFs catalyzed SBH hydrolysis with an (Ea) of 16.30 kJ mol^−1^. Since the (Ea) of the Ni_0.3_Co_0.5_Pd_0.2_ tri-NPs@CNFs catalyst was relatively low, the catalyst was able to generate hydrogen at a rapid rate. The superiority of the current Ni_0.3_Co_0.5_Pd_0.2_ tri-NPs@CNFs catalyst for H_2_ generation was demonstrated by comparing its catalytic performance to that of the reported articles in H_2_ production from various precursors using trimetallic NPs; the findings are displayed in Table 3.

## 7. Recyclability Studies of Ni_0.3_Co_0.5_Pd_0.2_ tri-NPs@CNFs Catalyst towards SBH Hydrolysis

**Figure 9:**
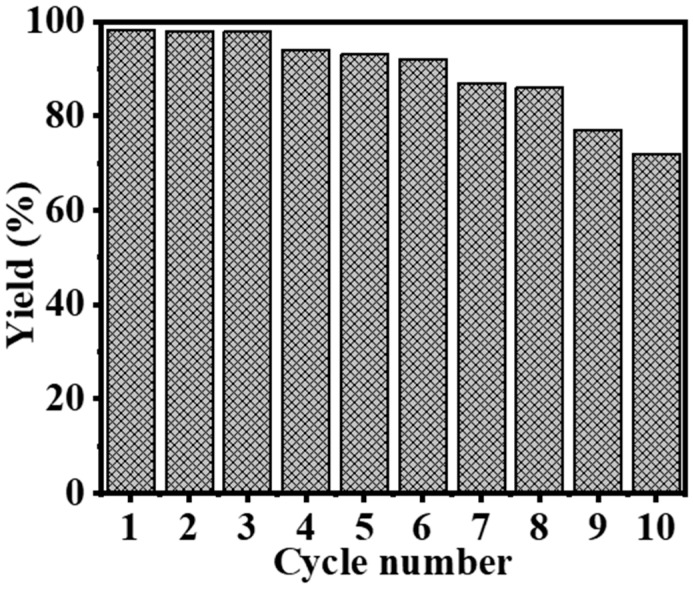
The reusability of the Ni_0.3_Co_0.5_Pd_0.2_@CNFs catalyst.

Commercial applications of heterogeneous catalysts are possible if the catalysts demonstrate both high activity and recyclability outcomes [19,57,58,59]. The results from a recyclability investigation of the Ni_0.3_Co_0.5_Pd_0.2_ tri-NPs@CNFs catalyst are presented in Figure 9. The hydrolysis of all stoichiometric H2 samples was shown in the reaction with new Ni_0.3_Co_0.5_Pd_0.2_ tri-NPs@CNFs catalysts at 25 °C in the presence of 1 mmol of SBH and 0.05 g of a Ni_0.3_Co_0.5_Pd_0.2_ tri-NPs@CNFs catalyst. In subsequent cycles, 1 mmol of SBH was added without washing or makeup the catalyst. The initial three cycles showed consistent catalytic activities. While following the third cycle, the catalytic activity gradually decreased from 94% in the fourth cycle to 72% in the tenth. The FeCuCo catalyst was kept at 80% of its initially catalytic activity after eight cycles [19]. The AC@Pt-Ru-Ni NP was reserved at 75% of its initially activity after three cycles [49]. CoBMo/Cu showed a good performance for five cycles, which kept 98% of its initial activity [60]. A decreased catalytic performance can be caused by an increase in solution viscosity, which reduces the number of available active sites or causes pores to become blocked due to the deposition of a sodium metaborate by-product [61,62,63,64,65]. Overall, the Ni_0.3_Co_0.5_Pd_0.2_ tri-NPs@CNFs catalyst has a significant recyclable performance of up to ten recycles and may be a suitable catalyst material for H_2_ production from the hydrolysis of SBH. 

## 8. Conclusions

Sol-gel and electrospinning approaches were used to prepare NiCoPd tri-NPs@CNFs catalysts. The hydrolysis process of SBH indicated that the utilization of the as-prepared NiCoPd tri-NPs@CNFs catalyst with a different composition presented superior catalytic activity towards the generation of H_2_. In comparison to the other formulations, it was demonstrated that the sample composed of Ni_0.2_Co_0.5_Pd_0.2_ tri-NPs@CNFs possessed the highest level of catalytic activity. Moreover, compared to the bimetallic Ni_0.5_Co_0.5_@CNFs (468.41 mol H_2_ per mol-M), the prepared NiCoPd tri-NPs@CNFs demonstrated greater activity for H_2_ generation (Ni_0.2_Co_0.5_Pd_0.2_ tri-NPs@CNFs, 866.42 mol-H_2_ per mol-M). In addition, the catalyst had a low activation energy of 16.40 kJ mol^−1^, which was remarkable in comparison to the other reported catalysts. It is interesting to note that the catalyst maintained a good activity for up to 10 recycles without any washing or makeup catalyst during the recyclability process, which proved its effectiveness and durability.

## Figures and Tables

**Table 1:** The H_2_ generation rate for the hydrolysis of 1 mmol of SBH at 25 °C catalyzed by different compositions of Ni/Co/Pd@CNFs.

Catalyst	Volume (mL)	Time (min)	Yield (%)	Rate (mL H_2_/min)	Rate (mol H_2_/h. mol Metal)
Ni_0.5_Co_0.5_	103	31	86	3.30	468
Ni_0.7_Co_0.1_Pd_0.2_	107	31	89	3.45	486
Ni_0.5_Co_0.3_Pd_0.2_	115	31	95.8	3.70	522
Ni_0.3_Co_0.5_Pd_0.2_	118	14	100	8.42	1188
Ni_0.1_Co_0.7_Pd_0.2_	118	21	100	5.60	792

**Table 2:** The H_2_ generation rate for hydrolysis of 1 mmol SBH at 25 °C catalyzed by different catalyst loading amounts of Ni/Co/Pd@CNFs.

Catalyst Loading (g)	Volume (mL)	Time (min)	Yield (%)	Rate (mL H_2_/min)
0.05	118	14	100	8.42
0.1	118	11	100	10.7
0.15	118	8	100	14.75
0.2	118	6	100	19.67

**Table 3:** The activation energies of trimetallic NPs in H_2_ production using different H_2_ sources.

Catalyst	E_a_ (KJ/mol)	Ref.
(Ni_5_Pt_5_)_1_-(CeO_x_)_0.3_/NGH	38.66	[53]
Ni_0.25_Fe_0.25_Pd_0.5_/UiO-66	43.5	[54]
Ni_45_Au_45_Co_10_	18.8	[2]
PdRuNi@GO	55.47	[55]
AC@Pt-Ru-Ni	24.29	[49]
Cu_0.04_Co_0.864_Ni_0.096_	40	[18]
Ru-capped/FeCo	42.9	[56]
Ni_0.3_Co_0.5_Pd_0_._2_	16.30	This study
